# Hepatocellular carcinoma detection via targeted enzymatic methyl sequencing of plasma cell-free DNA

**DOI:** 10.1186/s13148-022-01420-6

**Published:** 2023-01-04

**Authors:** Ping Guo, Hailing Zheng, Yihan Li, Yuntong Li, Yue Xiao, Jin Zheng, Xingqiang Zhu, Huan Xu, Zhi He, Qian Zhang, Jinchun Chen, Mingshan Qiu, Min Jiang, Pingguo Liu, Hongliang Chen

**Affiliations:** 1 School of Medicine, Huaqiao University, Xiamen, 361021 Fujian People’s Republic of China; 2 Xiamen Vangenes Biotechnology CO., LTD, Xiamen, 361015 Fujian People’s Republic of China; 3 Xiamen Hospital of Beijing University of Chinese Medicine, Xiamen, 361001 Fujian People’s Republic of China; 4 Zhongshan Hospital, Xiamen University, Xiamen, 361004 Fujian People’s Republic of China; 5 The Second Affiliated Hospital of Fujian University of Traditional Chinese Medicine, Fuzhou, 350003 Fujian People’s Republic of China; 6 School of Life Sciences, Xiamen University, Xiamen, 361102 Fujian People’s Republic of China

**Keywords:** Enzymatic methyl sequencing, Liquid biopsy, Hepatocellular carcinoma, Cancer screening, Incomplete Conversion

## Abstract

**Background:**

Epigenetic variants carried by circulating tumor DNA can be used as biomarkers for early detection of hepatocellular carcinoma (HCC) by noninvasive liquid biopsy. However, traditional methylation analysis method, bisulfite sequencing, with disadvantages of severe DNA damage, is limited in application of low-amount cfDNA analysis.

**Results:**

Through mild enzyme-mediated conversion, enzymatic methyl sequencing (EM-seq) is ideal for precise determination of cell-free DNA methylation and provides an opportunity for HCC early detection. EM-seq of methylation control DNA showed that enzymatic conversion of unmethylated C to U was more efficient than bisulfite conversion. Moreover, a relatively large proportion of incomplete converted EM-seq reads contains more than 3 unconverted CH site (CH = CC, CT or CA), which can be removed by filtering to improve accuracy of methylation detection by EM-seq. A cohort of 241 HCC, 76 liver disease, and 279 normal plasma samples were analyzed for methylation value on 1595 CpGs using EM-seq and targeted capture. Model training identified 283 CpGs with significant differences in methylation levels between HCC and non-HCC samples. A HCC screening model based on these markers can efficiently distinguish HCC sample from non-HCC samples, with area under the curve of 0.957 (sensitivity = 90%, specificity = 97%) in the test set, performing well in different stages as well as in serum α-fetoprotein/protein induced by vitamin K absence-II negative samples.

**Conclusion:**

Filtering of reads with ≥ 3 CHs derived from incomplete conversion can significantly reduce the noise of EM-seq detection. Based on targeted EM-seq analysis of plasma cell-free DNA, our HCC screening model can efficiently distinguish HCC patients from non-HCC individuals with high sensitivity and specificity.

**Supplementary Information:**

The online version contains supplementary material available at 10.1186/s13148-022-01420-6.

## Introduction

Hepatocellular carcinoma (HCC) is a common malignant tumor of the digestive system and one of the leading causes of tumor death worldwide [1, 2]. Symptoms in patients with early stage HCC are not quite obvious. Liver ultrasound and serum alpha-fetoprotein (AFP) examination are commonly used for HCC screening [3]. Randomized controlled trials have shown that improving early detection of HCC by monitoring hepatitis B virus carriers can significantly reduce HCC mortality [4, 5]. However, the sensitivity of AFP and liver ultrasound for early stage of HCC is relatively low [6, 7]. Therefore, there is an urgent need to develop a sensitive, reliable, and minimally invasive assay to detect early stage HCC for timely intervention.

Plasma cell-free DNA (cfDNA) refers to the degraded DNA fragments that are released into plasma after cell necrosis or apoptosis [8, 9]. In cancer patients, a portion of cfDNA is derived from the tumor cells, also known as circulating tumor DNA (ctDNA) [10, 11]. Next-generation sequencing (NGS) can analyze a variety of tumor-specific signals carried by the ctDNA, including somatic gene mutations [12], methylation modifications [13, 14], end motif [15], fragment length profiles [16], etc. Among them, methylation modification analysis is widely used for detection of HCC [17], colorectal cancer [18, 19], and even pan-cancer [20] due to its advantages of reflecting the early changes of tumors and the origin of tumor cells.

Bisulfite sequencing (BS-seq) is considered the gold standard for DNA methylation analysis because it provides quantification of methylation signals at single-base resolution [21]. However, the harsh conditions of the bisulfite treatment can cause huge damage to the DNA, which leads to generally poor sequence diversity, target enrichment bias, and also high sequencing errors [22, 23]. These shortcomings limit the application of BS-seq for methylation analysis of low-input DNA (e.g., cfDNA).

The limitation of BS-seq drives the development of new methylation detection techniques. TET-assisted pyridine borane sequencing (TAPS) utilizes the combination of biological enzymes and chemical methods to differentiate cytosine (C), 5-methylation of cytosine (5mC), and 5-hydroxymethylation of cytosine (5hmC) without damaging the DNA [24]. Enzymatic methyl-seq (EM-seq) uses enzymes including ten–eleven translocation dioxygenase 2 (TET2), T4 phage β-glucosyltransferase (T4-βGT), apolipoprotein B mRNA editing enzyme, catalytic polypeptide-like (APOBEC3A) to achieve the similar conversion to bisulfite treatment for methylation analysis. That is, unmethylated C is converted to T (Thymine), without any change in 5mC and 5hmC [25]. Compared with BS-seq, the EM-seq method based on mild biological enzyme conversion has obvious advantages including improved coverage and more even GC distribution [25].

In this study, we investigated incomplete conversion of EM-seq in hypomethylated control DNA and minimized the methylation signal noise by filtering the incomplete converted sequences. We confirmed the high sequencing quality of EM-seq for the analysis of low-amount DNA samples. Using EM-seq and target capture methods, a total of 596 clinical plasma cfDNA samples were analyzed in our study to develop an HCC screening model with good performance.

## Results

### Profiling and minimizing incomplete conversion of EM-seq

**Fig. 1:**
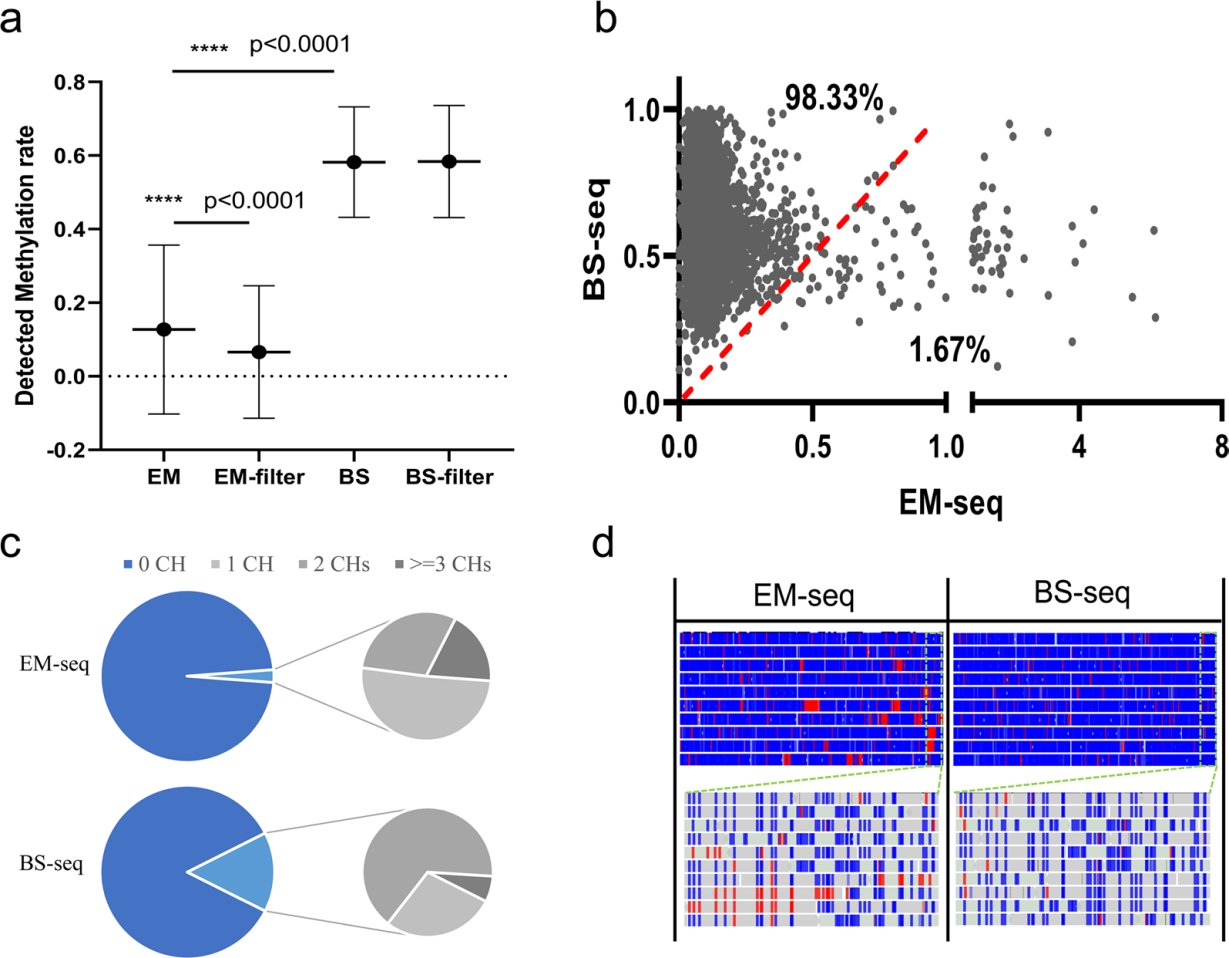
In complete conversion in EM-seq. **a** Methylation values on hypomethylated CpGs acquired by EM-Seq and BS-seq, before and after ≥ 3CHs filtration. Hypomethylated CpGs are those with BS-seq detection values of < 1% on λ DNA. **b** Dot plot compares individual methylation values in hypomethylated CpGs acquired by EM-Seq and BS-seq. Percentages indicate the fraction of CpGs that differed between conditions. **c** Pie charts compare the proportion of reads with 0, 1, 2, and ≥ 3 CH sites in all EM-seq and BS-seq sequencing reads. d. Genome plot for unmethylated control λ DNA (pos: 30,000–35,000) compares CH reads between EM-seq and BS-seq datasets. Boxes represent reads, and unmethylated (blue) and methylated (red) CHHs are indicated

To investigate the performance of EM-seq, we used it to analyze methylated and unmethylated DNA control and compared with BS-seq. Among hypermethylated CpG sites, the methylation value detected by EM-seq was comparable to that by BS-seq (Additional file 1: Fig. S1a). Among the hypomethylated CpG sites, the methylation value detected by EM-seq was significantly lower than that by BS-seq (Fig. 1a, 0.1% V.S. 0.59%, *P* value < 0.0001). However, approximately 1.67% of hypomethylated CpGs (103 CpGs) had higher detection values in EM-seq than in BS-seq (Fig. 1b), and abnormal hypermethylation signals of > 1% were observed at 20 CpGs in EM-seq, accompanied by hypermethylation signals at adjacent CH sites (CH = CC, CT or CA, Additional file 2: Table S1). This suggests that EM-seq is prone to incomplete conversion in specific context, resulting in methylation signal noise, probably due to the substrate specificity of APOBEC3A deamination [26].

Non-CG methylation is rare in most human cells and can therefore be used as an internal unmethylated control for methylation detection [27]. To further investigate the incomplete conversion, reads containing CH were extracted from BS-seq and EM-seq data. More CH reads were observed in BS-seq data than in EM-seq data (Fig. 1c). Both sporadic and clustered CHs were observed in EM-seq, and the reads with ≥ 3 CHs accounted for 18.71% of all CH reads in EM-seq (Fig. 1c, d, Additional file 1: Fig. S1b). In contrast, CH was mostly distributed sporadically in BS-seq, and the proportion of ≥ 3 CHs reads was only 6.43% of CH reads in BS-seq (Fig. 1c, d, Additional file 1: Fig. S1b). A larger proportion of ≥ 3 CHs reads was also observed from EM-seq data than BS-seq data from previous study with human genomic DNA (Additional file 1: Fig. S1c) [25].

We next considered if the performance of EM-seq could be improved by filtering reads with incomplete conversion. Filtering reads with ≥ 3 CHs significantly reduced EM-seq value of hypomethylated CpGs while not affect that of hypermethylated CpGs (Fig. 1a, Additional file 1: Fig. S1d). In contrast, filtration had limited impact on the result of BS-seq (Fig. 1a, b), consistent with previous report [28]. Furthermore, the number of CG sites with EM-seq values higher than BS-seq values decreased by approximately 45.5% (Additional file 1: Fig. S1e), suggesting that filtering of ≥ 3CHs reads can further improve the accuracy of EM-seq.

### Methylation marker selection and targeted EM-seq

**Fig. 2:**
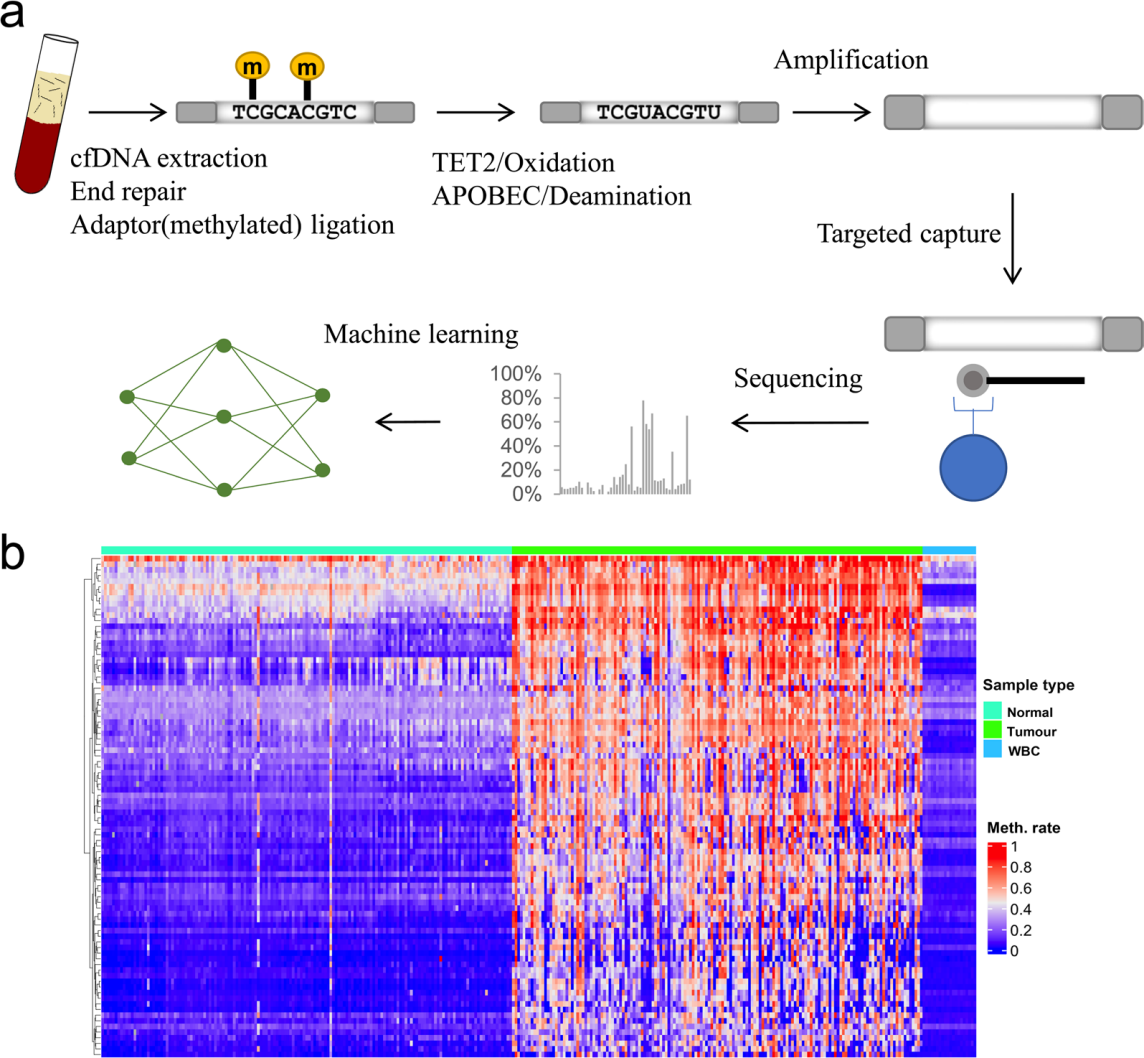
Targeted EM-seq and marker selection. **a** Overview of targeted EM-seq of plasma cell-free DNA. **b** Unsupervised hierarchical clustering of 89 methylation markers selected for panel design

Because deep sequencing of the whole human methylome would be prohibitively costly, we used EM-seq combined with targeted capture to analyze plasma cfDNA from HCC patients and non-HCC controls (Fig. 2a). Briefly, plasma DNA was ligated to the methylated adaptor before enzymatic conversion and amplified using primers with dual index. Multiple samples were pooled together and enriched for target region by the designed probe panel before deep sequencing.

To find potential biomarkers for HCC detection, we analyzed the expression profile data and methylation data of HCC and non-HCC tissues and screened CpGs with relatively high methylation from low-expression genes in HCC. Then, the methylation data of whole blood DNA were used to eliminate the CpGs whose average methylation level in blood was higher than that in HCC (Fig. 2b). To cover the CpGs associated with HCC as much as possible, other CpGs suitable for HCC detection were also included in our analysis, according to previous studies [29–36]. Finally, we designed a probe panel covering 1595 CpGs for subsequent analysis.

We used HepG2 cell DNA and plasma DNA to evaluate the performance of the targeted EM-seq. Relatively high DNA recovery was achieved in targeted EM-seq, with 60–70% of reads uniquely aligned to the designed panel region (target ratio) and median unique read depth of over 800 × with 20 ng fragmented cell DNA (Additional file 1: Fig. S2a, Additional file 2: Table S2). In addition, we confirmed the consistency of methylation levels detected by two Targeted EM-seq technical repeats (Additional file 1: Fig. S2b) along with the consistency of coverage depth (Additional file 1: Fig. S2c). EM-seq analysis of a representative donor cfDNA showed the length characteristics of cfDNA including the main peak of about 167 bp and small peaks spaced by 10 bp (Additional file 1: Fig. S2d) [37].

### HCC detection using plasma samples

**Fig. 3:**
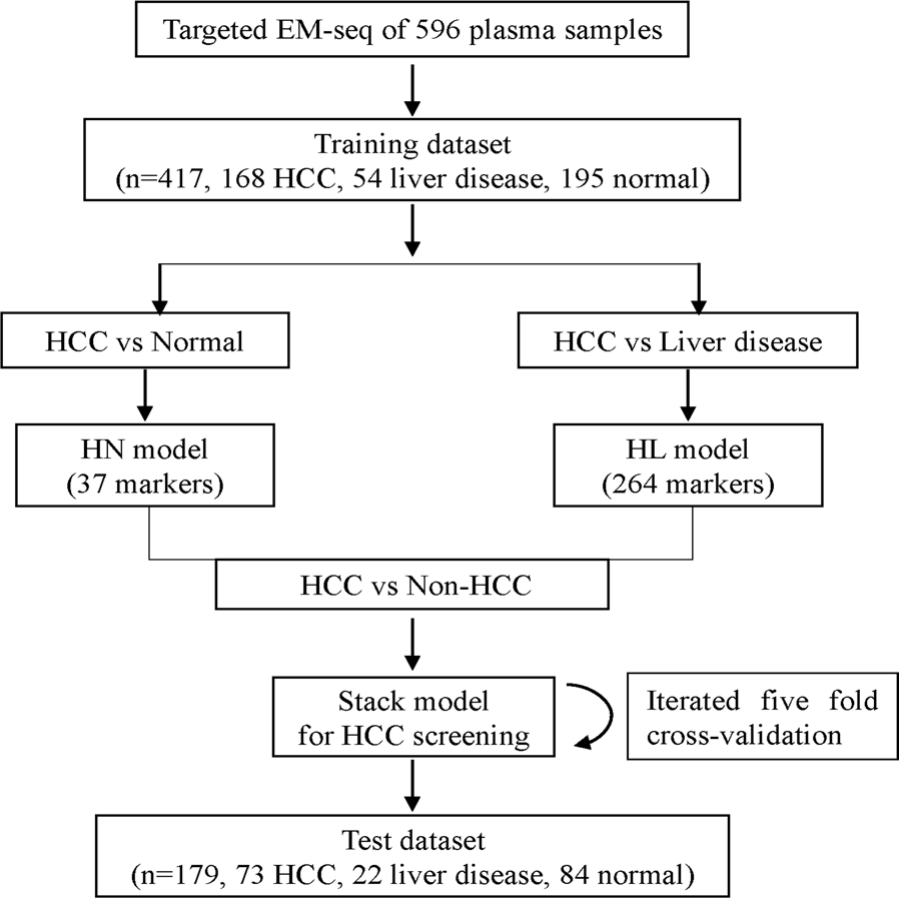
Workflow chart of building a stack HCC screening model

To develop a predictive model for the HCC screening, 596 plasma samples were analyzed by Targeted EM-seq (Additional file 2: Table S3). These samples were randomly divided into training set (*n* = 417, normal controls = 195, liver disease = 54, HCC = 168) for the model training; and test set (*n* = 179, normal controls = 84, liver disease = 22, HCC = 73) for the model testing. We confirmed the consistency of methylation levels of 20 plasma samples detected by two technical repeats of hybridization capture (Additional file 2: Table S4, *r* > 0.99). The study design is depicted in Fig. 3. Detailed patient characteristics are summarized in Additional file 2: Table S5.

**Fig. 4:**
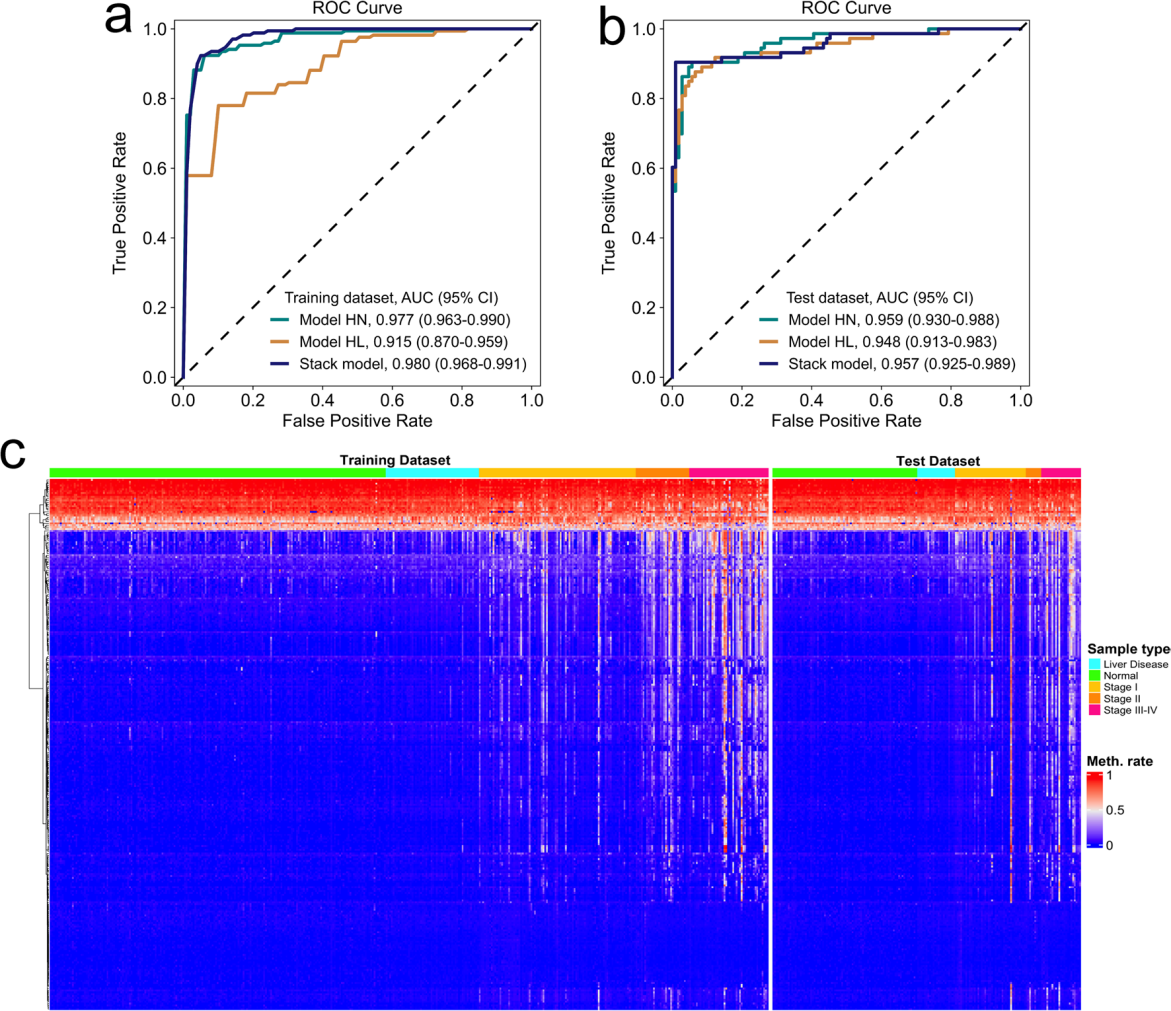
Development and validation of the HCC screening model. **a**, **b** ROC of the three models with methylation markers in the training dataset (**a**) and test dataset (**b**). **c** Unsupervised hierarchical clustering of 283 methylation markers selected for HCC screening model development in the training and test datasets

GDBT (gradient boosting decision tree) machine learning analyses were applied to the training cohort of 168 HCC and 195 normal controls to generate a HCC versus normal model (HN model) with 37 markers. HN model achieved a sensitivity of 87% and a specificity of 97% ( area under the curve, AUC = 0.977) in the training set and a sensitivity of 90% and a specificity of 94% (AUC = 0.959) in the test set (Fig. 4a). The same analysis of 168 HCC and 54 liver diseases obtained a HCC versus liver disease model (HL model) with 264 markers. HL model achieved a sensitivity of 90% and a specificity of 72% (AUC = 0.915) in the training set and a sensitivity of 92% and a specificity of 84% (AUC = 0.948) in the test set (Fig. 4b).

A total of 283 markers were obtained after the two sets of markers were combined and deduplicated (Additional file 2: Table S6). The methylation levels of these markers were significantly different between HCC and non-HCC samples in both training and test sets, and the intensity increased with advancing disease stage (Fig. 4c, Additional file 2: Table S7).

**Fig. 5:**
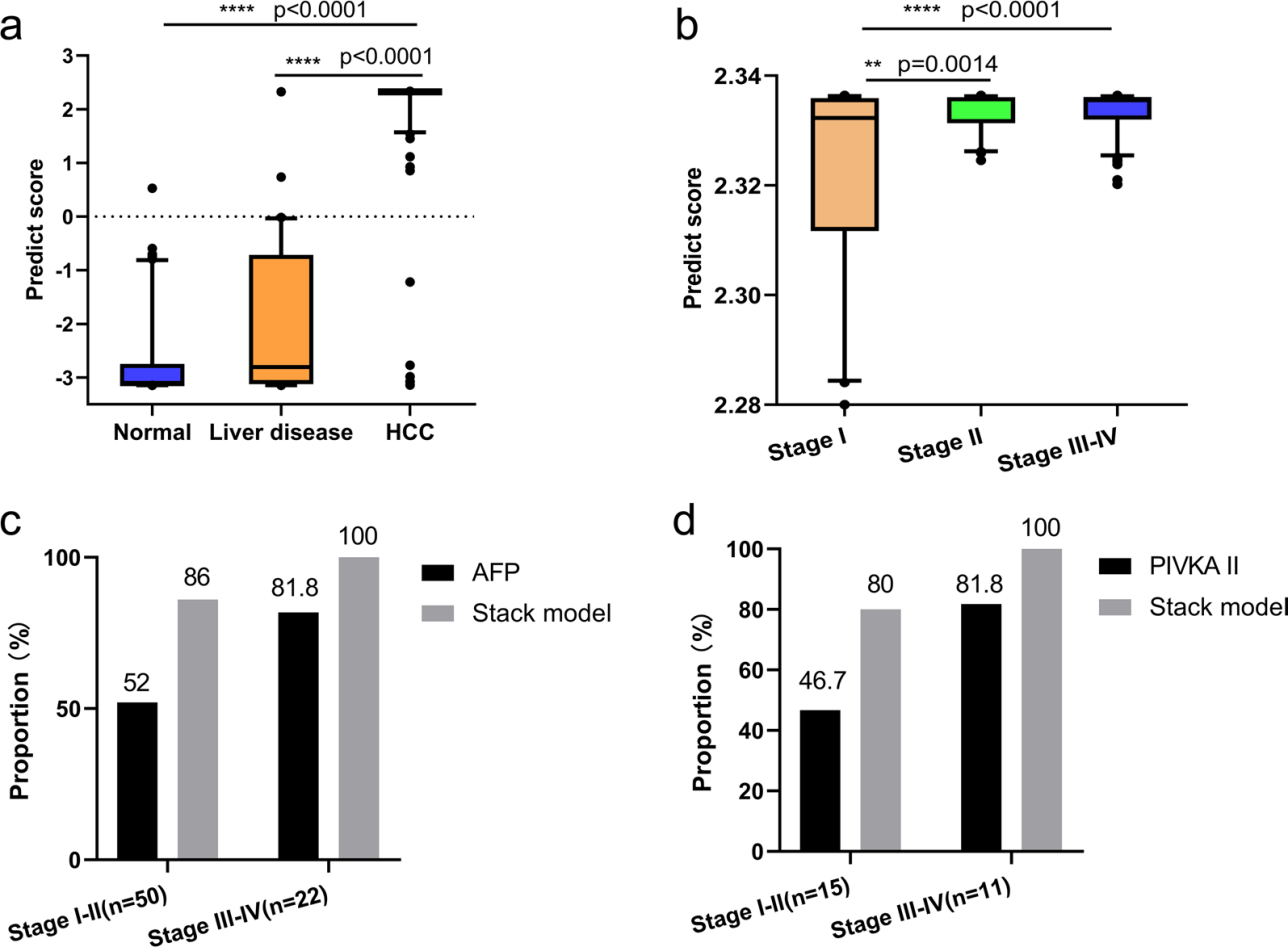
Further evaluation of the stack model for HCC screening. **a** The t-score of the stack model in normal controls, individuals with liver diseases and HCC patients. **b** The t-score in HCC patients with early and advanced stages. **c** Proportions of positive calling by the stack model and AFP (> 20 in HCC patients with early and advanced stages in the test set). **d** Proportions of positive calling by the stack model and PIVKA-II in HCC patients with early and advanced stages in the test set

Using a logistic regression method, we next constructed a stack model for HCC screening with these 283 markers using the predictive values generated from HN and HL models. Overall, the stack model yielded a sensitivity of 92% and a specificity of 94% in the training/fivefold cross-validation set (AUC = 0.980, 95% confidence interval [CI] 0.968–0.991; Fig. 4a), and a sensitivity of 90% and a specificity of 97% in the test set (AUC = 0.957, 95% CI 0.925–0.989; Fig. 4b). The classification accuracy was highly consistent between these two sets (AUC 0.980 versus 0.957), which confirms appropriate control of overfitting risks. HCC tumor score (t-score), the outputs of the stack model, can efficiently distinguish HCC patients from non-HCC individuals (Fig. 5a).

### Subgroup analysis and comparison with AFP/PIVKA-II

The stack model for HCC screening achieved high sensitivity and specificity in different groups. Sensitivity improved with advancing disease stage and achieved 85% in stage I (35/41), 89% in stage II (8/9), and 100% in stage III-IV (23/23) patients in the test set (Table 1). Similarly, there is good correlation between the t-score and tumor stage. Patients with early stage disease (I, II) had substantially lower t-scores compared to those with advanced stage disease (III, IV) (Fig. 5b). The specificity was 99% for normal controls (1/84) and 91% for liver disease samples (2/22) in test set (Table 1).

**Table 1:** Performance of the stack model for HCC screening in the test set

Groups	Total	Predicted		Sensitivity (%)	Specificity (%)
		Non-HCC	HCC		
Healthy	84	83	1		99
Liver disease	22	20	2		91
Non-HCC	106	103	3		97
I	41	6	35	85	
II	9	1	8	89	
III-IV	23	0	23	100	
HCC	73	7	66	90	

In HCCs, the stack model achieved higher detection accuracy than AFP for both early and advanced HCC (Fig. 5c) and achieved high detection accuracy in AFP-negative patients (25 of 28 patients for AFP < 20 ng/mL, 89.29%, Additional file 1: Fig. S3a). Protein induced by vitamin K absence-II (PIVKA-II) is another potential screening marker for HCC [38]. The stack model also achieved higher detection accuracy than PIVKA-II for both early and advanced HCC (Fig. 5d) and achieved high detection accuracy in PIVKA-II-negative patients (8 of 10 patients for PIVKA-II < 40 ng/mL, 80.0%) (Additional file 1: Fig. S3b).

## Discussion

Diagnosis of HCC patients at early stage can effectively reduce HCC mortality. Tumor-specific methylation in cell-free DNA provides the possibility for noninvasive liquid biopsy of tumor detection and monitoring [10, 39, 40]. In this study, we used the targeted EM-seq method, replacing the traditional BS-seq, for methylation analysis of the plasma DNA from HCC patients and non-HCC controls, and constructed a highly sensitive and specific HCC screening model.

EM-seq has significant advantages over BS-seq. First, compared with BS-seq, EM-seq result in less DNA loss than BS-seq, achieving better sequencing coverage, especially in the analysis of low-input DNA samples. Second, EM-seq contains fewer conversion errors than BS-seq. In particular, our data in this study showed that the incomplete converted CHs in EM-seq cluster in one read, which can be easily removed by filtering. Minimizing the methylation noise caused by the incomplete conversion is important to accurately detect trace methylation variation from limited ctDNA in early stage samples [41]. Finally, EM-seq library can be prepared by ligating the adaptor before conversion without obvious DNA loss. The library obtained by this method can retain more physiological characteristics of cfDNA and has the potential to analyze methylation, end motif, and fragmentation profiles simultaneously.

Due to the substrate specificity of APOBEC3A deamination, incomplete conversion of EM-seq occurs in a few specific context, leading to false increase of > 1% in methylation rate. Including APOBEC enzymes with sequence specificity other than APOBEC3A may further improve conversion efficiency of EM-seq. Besides, sequence-specific incomplete conversion in a few specific sites can offset each other across clinical samples during biomarker selection. So it does not deny the results of this study.

The HCC screening model constructed by machine learning analysis can clearly distinguish HCC and non-HCC samples with 90% sensitivity and 99% specificity in the test cohort. Cancer is a rare disease, and the incidence of HCC is low in the population. Only extremely specific tumor screening methods can minimize the risk of overdiagnosis and achieve effective screening for the HCC [42]. In addition, the HCC screening model in this study has different sensitivities to HCC samples of different stages. The sensitivity of advanced stage samples is higher than that of early stage samples, indicating that tumor burden has a certain influence on the prediction results of the HCC screening model. This model holds potential for monitoring of HCC treatment.

This study has some limitations as well. First, fewer liver disease samples were recruited in this study and the inclusion of more high-risk samples would help to clarify the specificity of the screening model in high-risk populations. Second, the median age of the non-HCC control group in this study was 46 years, which was lower than that of the HCC group (median 57 years). Methylation is correlated with age [43]. Therefore, the inclusion of age-matched non-HCC controls helps to eliminate the interference of age-related methylation variation.

## Conclusions

This study demonstrated that incomplete conversion of EM-seq is characterized by CH clustering in sequencing reads. Filtering of reads with ≥ 3 CHs can significantly reduce the noise of methylation detection of EM-seq. A predictive model for HCC screening based on targeted EM-seq analysis of plasma cell-free DNA can efficiently distinguish HCC patients from non-HCC individuals and perform well in the detection of early stage patients. It provides a noninvasive method for HCC screening with a very high specificity. Given that epigenetic variations are common in the development of most tumors, this strategy could be modified for the early detection of other cancer types or of multiple cancer types from a single tube of blood.

## Methods

### Study design

Patients with HCC or liver disease that were treated at the Zhongshan Hospital, Xiamen University, between November 2020 and December 2021 were selected for this study. The HCC stage was determined using the China liver cancer staging system [44]. Healthy individuals undergoing routine health care maintenance were randomly selected as controls in this study. Detailed patient characteristics are summarized in Additional file 2: Table S1. The present study was performed under the Helsinki Declaration and was approved by the Ethics Committee of the Zhongshan Hospital, Xiamen University (reference number: 2020-015). Informed consent was obtained from all participants or their families.

### Blood sample processing and cfDNA purification

Samples from all cases and controls were processed by the following method. Peripheral blood was collected in cfDNA Blood Collection Tube (Zhixuan Biotech). Plasma was separated by centrifugation at 1600×*g* for 10 min and transferred to microcentrifuge tubes. After then, centrifugation was done at 16,000×*g* for 10 min to remove cellular debris. The supernatant was divided into 2-ml aliquots and stored at − 80 °C until the time of DNA extraction. cfDNA was extracted from 2-ml plasma for each participant using Plasma Cell-Free DNA Extraction Kit (Concert). cfDNA concentration was measured using Qubit dsDNA High Sensitivity Assay Kit (Thermo Fisher).

### Enzymatic methyl conversion

EM-seq library preparation was performed using VAHTS Universal DNA library Prep Kit for Illumina V3 (Vazyme) and EM-seq Conversion Module (New England BioLabs) according to the manufacturer’s instruction with minor modifications. In brief, mechanically fragmented methylation control DNA (CpG methylated pUC19 and unmethylated λ DNA) alone or combined with cell-free DNA fragments was treated with VAHTS Universal DNA library Prep Kit (New England BioLabs) for end-repair, A-tailing, and ligation of EM-seq adaptor (New England BioLabs). The ligated samples were methyl-converted with EM-seq Conversion Module (New England BioLabs) per the manufacturer’s protocol. Methyl-converted DNA was purified and amplified using NEBNext Unique Dual Index Primers (New England BioLabs) and KAPA HiFi HotStart Uracil + ReadyMix (KAPA biosystems).

### Bisulfite treatment

Methylation control DNA or DNA fragments were ligated with the EM-seq adaptor as mentioned above and then subjected to the bisulfite conversion using EZ DNA Methylation-Lightning Kit (Zymo Research) according to the manufacturer’s protocol.

### Targeted capture and sequencing

EM-seq libraries from up to 24 samples were pooled together for hybridization enrichment using the Customized NAD probes (Nanodigmbio). The capture reaction was performed with Nadprep Hybrid capture Reagents (Nanodigmbio) following the manufacturer’s instructions. Captured libraries were obtained using on-bead PCR amplification with VAHTS HiFi Amplification mix and PCR primer mix3 for Illumina (Vazyme). The libraries size was determined using the Bioanalyzer 2100 (Agilent Technologies). The target-enriched library was then sequenced on the NovaS4 NGS platform (2 × 150 bp, Illumina) following the manufacturer’s instructions.

### Panel design

Methylation and expression profile data of 357 HCC and 50 non-HCC tissue were downloaded from The Cancer Genome Atlas (TCGA) [45]. Differential analysis was performed both on expression profiles of all genes except for those on sex chromosomes and methylation data of the gene-related CpG islands. The screening thresholds for differential gene expression were set as FDR < 0.05 and |log2 (fold change)|> 1. The screening thresholds for differential methylation were set as FDR < 0.05 and |β|> 0.2, and the methylation sites with high methylation and low expression were selected. The methylation dataset GSE69270 of 184 non-HCC blood was downloaded from the Expression Omnibus database [46, 47]. The CpG sites with an average methylation level in non-HCC blood higher than that in HCC tissues (> 0.1) were removed. A group of five probes of 120 bp length was designed for each CpG site, including NO.1 the original unconverted sequence, NO.2 and NO.3 the converted, methylated Watson and Crick strands, NO.4 and NO.5 the converted, unmethylated Watson and Crick strands. Multiple probes were used to reduce the effect of the original methylation state on the capture efficiency.

### Data processing

The raw sequencing FASTQ files were processed using Fastp (0.21.0) to trim Illumina-specific adapters with default parameters and low-quality sequences with parameters of -u 20 and -q 20 [48]. Mapping of the processed sequencing reads was performed using the bismark (0.23.0) [49]. Deduplicate_bismark (bismark) was used for deduplication. Incomplete converted reads with more than 3 CHs were removed. Finally, methylation level is estimated using bismark_methylation_extractor (bismark) to calculate methylation frequencies for all CpGs using parameters -comprehensive --bedGraph --counts --cytosine_report --CX --buffer_size 20G --parallel 16.

### Development of HCC screening model

A custom module was built to classify samples using two layers of models: (i) two GDBT model: A HCC versus normal model (HN model) and a HCC versus liver disease (HL model) [50]. Both models were trained by GDBT model. RFECV (recursive feature elimination with cross-validation) method was used to select the optimal features in HN model [51]. In HL model, all features with importance greater than 0 were selected as the optimal features. GridSearchCV (grid search with cross-validation) method was used to optimize models’ parameters [52]. (ii) A multinomial logistic regression model: two models of the first layer were stacked into a logistic regression model to obtain a HCC/non-HCC assignment as a final prediction [53]. The predictive score *h* (*t*-score) is calculated as: *h* = *w*^*T*^*x* + *b*, where *w* is the coefficient of each feature, and *x* is the output from models in the first layer. The L2 penalty parameter *λ* was determined by GridSearchCV method. To optimize model performance and avoid overfitting, iterative fivefold cross-validation was performed in the training dataset. The performance of the final stacked model was evaluated in the independent test cohort.

### Statistical analysis

Differences between the groups were calculated using the Student’s t test. Statistical analysis was done by R (v. 4.0.5). The performance of prediction model was evaluated using the AUC statistics (95% CI), and the values were calculated by the pROC (v. 1.18.0) package [54]. The sensitivity [True Positive/(True Positive + False Negative)] and specificity [True Negative/(True Negative + False Positive)] thresholds under 95% CI are obtained by the formula: $p \pm 1.96 \times \sqrt {p\left( {1 - p} \right)/n}$ [55].

## Supplementary Information

**Additional file 1: Figure S1.** Comparison of conversion efficiency between EM-seq and BS-seq. **a** Dot plot compare individual methylation values in hypermethylated CpGs acquired by EM-Seq and BS-seq (*r* = 0.91, *P* < 0.0001). Percentages indicate the fraction of CpGs that differed between conditions. Hypermethylated CpGs are those with BS-seq detection values of > 80% on pUC19 DNA. **b** Genome plot for unmethylated control λ DNA(30,000–35,000)compares CH reads between EM-seq and BS-seq datasets. Boxes represent reads, and unmethylated (blue) and methylated (red) CHGs are indicated. **c** Pie charts compare the proportion of reads with 0, 1, 2 and ≥ 3 CH sites in EM-seq (SRR10532128) and BS-seq (SRR10532135) sequencing reads from public database. **d** Methylation values on hypermethylated CpGs acquired by EM-Seq and BS-seq, before and after ≥ 3CHs filtration. ns represents no significance. **e** Dot plot compare individual methylation values in hypomethylated CpGs acquired by EM-Seq and BS-seq after 3CH read filtering. Percentages indicate the fraction of CpGs that differed between conditions. **Figure S2.** Performance of targeted EM-seq. **a** Unique read depth (PCR duplication removed) observed in EM-seq and BS-seq with same input quantities (20 ng). **b** Heat maps compare individual CpG methylation values in the target panel acquired by targeted EM-Seq between technical replicates (*r* = 0.999). **c** Heat map shows coverage depth of CpGs between replicates of a 20 ng targeted EM-seq library (*r* = 0.982). **d** Fragment size distributions of plasma sample from a healthy donor. **Figure S3.** Comparison of HCC predictive model with AFP and PIVKA-II. **a** Proportions of positive calling by HCC screening model in HCC patients with different AFP levels in the test set. **b** Proportions of positive calling by HCC screening model in HCC patients with different PIVKA-II levels in the test set.

**Additional file 2**: **Table S1.** C sites prone to incomplete conversion in EM-seq. **Table S2**. Quality control metrics for Targeted EM-seq using HCC cell line input from 5 to 20 ng. **Table S3**. Quality control metrics for Targeted EM-seq from 596 clinical samples. **Table S4**. Comparison of methylation levels detected from two hybridization batches. **Table S5**. Summary of patient characteristics. **Table S6.** Summary of 283 CpG markers. **Table S7.** Methylation levels of 283 CpGs from clinical samples.

## Data Availability

The datasets supporting the conclusions of this article are included within the article and its additional files. All other datasets used and analyzed during the study are available from the corresponding author on reasonable request.

## Abbreviations

HCC: Hepatocellular carcinoma
AFP: Alpha-fetoprotein
cfDNA: Cell-free DNA
ctDNA: Circulating tumor DNA
NGS: Next-generation sequencing
BS-seq: Bisulfite sequencing
TAPS: TET-assisted pyridine borane sequencing
C: Cytosine
5mC: 5-Methylation of cytosine
5hmC: 5-Hydroxymethylcytosine
EM-seq: Enzymatic methyl-seq
TET2: Ten–eleven translocation dioxygenase 2
T4-βGT: T4 phage β-glucosyltransferase
APOBEC3A: Apolipoprotein B mRNA editing enzyme, catalytic polypeptide-like
T: Thymine
GDBT: Gradient boosting decision tree
AUC: Area under the curve
CI: Confidence interval
t-score: Tumor score
PIVKA-II: Protein induced by vitamin K absence-II
RFECV: recursive feature elimination with cross-validation

## References

[1] Zheng R, Qu C, Zhang S (2018). Liver cancer incidence and mortality in China: temporal trends and projections to 2030. Chin J Cancer Res.

[2] Bray F, Ferlay J, Soerjomataram I, Siegel RL, Torre LA, Jemal A (2018). Global cancer statistics 2018: GLOBOCAN estimates of incidence and mortality worldwide for 36 cancers in 185 countries. CA Cancer J Clin.

[3] Nguyen MH, Keeffe EB (2002). Screening for hepatocellular carcinoma. J Clin Gastroenterol.

[4] Zhang B-H, Yang B-H, Tang Z-Y (2004). Randomized controlled trial of screening for hepatocellular carcinoma. J Cancer Res Clin Oncol.

[5] Singal AG, Pillai A, Tiro J (2014). Early detection, curative treatment, and survival rates for hepatocellular carcinoma surveillance in patients with cirrhosis: a meta-analysis. PLoS Med.

[6] Marrero JA, Feng Z, Wang Y (2009). α-fetoprotein, des-γ carboxyprothrombin, and lectin-bound α-fetoprotein in early hepatocellular carcinoma. Gastroenterology.

[7] Singal A, Volk ML, Waljee A (2009). Meta-analysis: surveillance with ultrasound for early-stage hepatocellular carcinoma in patients with cirrhosis. Aliment Pharmacol Ther.

[8] Stroun M, Lyautey J, Lederrey C, Olson-Sand A, Anker P (2001). About the possible origin and mechanism of circulating DNA: apoptosis and active DNA release. Clin Chim Acta.

[9] Jahr S, Hentze H, Englisch S (2001). DNA fragments in the blood plasma of cancer patients: quantitations and evidence for their origin from apoptotic and necrotic cells. Cancer Res.

[10] Corcoran RB, Chabner BA (2018). Application of cell-free DNA analysis to cancer treatment. N Engl J Med.

[11] Roninson IB, Broude EV, Chang B-D (2001). If not apoptosis, then what? Treatment-induced senescence and mitotic catastrophe in tumor cells. Drug Resist Updat.

[12] Shu Y, Wu X, Tong X (2017). Circulating tumor DNA mutation profiling by targeted next generation sequencing provides guidance for personalized treatments in multiple cancer types. Sci Rep.

[13] Wen L, Li J, Guo H (2015). Genome-scale detection of hypermethylated CpG islands in circulating cell-free DNA of hepatocellular carcinoma patients. Cell Res.

[14] Xu R, Wei W, Krawczyk M (2017). Circulating tumour DNA methylation markers for diagnosis and prognosis of hepatocellular carcinoma. Nat Mater.

[15] Jiang P, Sun K, Peng W (2020). Plasma DNA end-motif profiling as a fragmentomic marker in cancer, pregnancy, and transplantation. Cancer Discov.

[16] Cristiano S, Leal A, Phallen J (2019). Genome-wide cell-free DNA fragmentation in patients with cancer. Nature.

[17] Wong IH, Lo YD, Zhang J (1999). Detection of aberrant p16 methylation in the plasma and serum of liver cancer patients. Cancer Res.

[18] Oh T, Kim N, Moon Y (2013). Genome-wide identification and validation of a novel methylation biomarker, SDC2, for blood-based detection of colorectal cancer. J Mol Diagn.

[19] Payne SR (2010). From discovery to the clinic: the novel DNA methylation biomarker m SEPT9 for the detection of colorectal cancer in blood. Epigenomics.

[20] Kisiel JB, Dukek BA, VSR Kanipakam R (2019). Hepatocellular carcinoma detection by plasma methylated DNA: discovery, phase I pilot, and phase II clinical validation. Hepatology.

[21] Krueger F, Kreck B, Franke A, Andrews SR (2012). DNA methylome analysis using short bisulfite sequencing data. Nat Methods.

[22] Tanaka K, Okamoto A (2007). Degradation of DNA by bisulfite treatment. Bioorg Med Chem Lett.

[23] Olova N, Krueger F, Andrews S (2018). Comparison of whole-genome bisulfite sequencing library preparation strategies identifies sources of biases affecting DNA methylation data. Genome Biol.

[24] Liu Y, Siejka-Zielińska P, Velikova G (2019). Bisulfite-free direct detection of 5-methylcytosine and 5-hydroxymethylcytosine at base resolution. Nat Biotechnol.

[25] Vaisvila R, Ponnaluri VC, Sun Z (2021). Enzymatic methyl sequencing detects DNA methylation at single-base resolution from picograms of DNA. Genome Res.

[26] Silvas TV, Hou S, Myint W (2018). Substrate sequence selectivity of APOBEC3A implicates intra-DNA interactions. Sci Rep.

[27] Lister R, Pelizzola M, Dowen RH (2009). Human DNA methylomes at base resolution show widespread epigenomic differences. Nature.

[28] Cokus SJ, Feng S, Zhang X (2008). Shotgun bisulphite sequencing of the Arabidopsis genome reveals DNA methylation patterning. Nature.

[29] Oussalah A, Rischer S, Bensenane M (2018). Plasma mSEPT9: a novel circulating cell-free DNA-based epigenetic biomarker to diagnose hepatocellular carcinoma. EBioMedicine.

[30] Wei L, Huang Y, Zhao R (2018). Detection of promoter methylation status of suppressor of cytokine signaling 3 (SOCS3) in tissue and plasma from Chinese patients with different hepatic diseases. Chin J Cancer Res.

[31] An Y, Guan Y, Xu Y (2019). The diagnostic and prognostic usage of circulating tumor DNA in operable hepatocellular carcinoma. Am J Transl Res.

[32] Cozma A, Fodor A, Vulturar R (2019). DNA methylation and micro-RNAs: the most recent and relevant biomarkers in the early diagnosis of hepatocellular carcinoma. Medicina.

[33] Wu H-C, Yang H-I, Wang Q, Chen C-J, Santella RM (2017). Plasma DNA methylation marker and hepatocellular carcinoma risk prediction model for the general population. Carcinogenesis.

[34] Tao L-P, Fan X-P, Fan Y-C, Zhao J, Gao S, Wang K (2018). Combined detection of insulin-like growth factor-binding protein 7 promoter methylation improves the diagnostic efficacy of AFP in hepatitis B virus-associated hepatocellular carcinoma. Pathol Res Pract.

[35] Huang Y, Wei L, Zhao R-C (2018). Predicting hepatocellular carcinoma development for cirrhosis patients via methylation detection of heparocarcinogenesis-related genes. J Cancer.

[36] Tian M-M, Fan Y-C, Zhao J (2017). Hepatocellular carcinoma suppressor 1 promoter hypermethylation in serum. A diagnostic and prognostic study in hepatitis B. Clin Res Hepatol Gastroenterol.

[37] Snyder MW, Kircher M, Hill AJ, Daza RM, Shendure J (2016). Cell-free DNA comprises an in vivo nucleosome footprint that informs its tissues-of-origin. Cell.

[38] Liebman HA, Furie BC, Tong MJ (1984). Des-γ-carboxy (abnormal) prothrombin as a serum marker of primary hepatocellular carcinoma. N Engl J Med.

[39] Pessoa LS, Heringer M, Ferrer VP (2020). ctDNA as a cancer biomarker: a broad overview. Crit Rev Oncol Hematol.

[40] Elshimali YI, Khaddour H, Sarkissyan M, Wu Y, Vadgama JV (2013). The clinical utilization of circulating cell free DNA (CCFDNA) in blood of cancer patients. Int J Mol Sci.

[41] Abbosh C, Birkbak NJ, Wilson GA (2017). Phylogenetic ctDNA analysis depicts early-stage lung cancer evolution. Nature.

[42] Srivastava S, Koay EJ, Borowsky AD (2019). Cancer overdiagnosis: a biological challenge and clinical dilemma. Nat Rev Cancer.

[43] Fraga MF, Agrelo R, Esteller M (2007). Cross-talk between aging and cancer: the epigenetic language. Ann N Y Acad Sci.

[44] Zhou J, Sun H, Wang Z (2020). Guidelines for the diagnosis and treatment of hepatocellular carcinoma (2019 Edition). Liver Cancer.

[45] Weinstein JN, Collisson EA, Mills GB (2013). The cancer genome atlas pan-cancer analysis project. Nat Genet.

[46] Edgar R, Domrachev M, Lash AE (2002). Gene expression omnibus: NCBI gene expression and hybridization array data repository. Nucleic Acids Res.

[47] Kananen L, Marttila S, Nevalainen T (2016). Aging-associated DNA methylation changes in middle-aged individuals: the Young Finns study. BMC Genomics.

[48] Chen S, Zhou Y, Chen Y, Gu J (2018). fastp: an ultra-fast all-in-one FASTQ preprocessor. Bioinformatics.

[49] Krueger F, Andrews SR (2011). Bismark: a flexible aligner and methylation caller for Bisulfite-Seq applications. Bioinformatics.

[50] Mason L, Baxter J, Bartlett P, Frean M. Boosting algorithms as gradient descent. Adv Neural Inf Process Syst. 1999; 12.

[51] Misra P, Yadav AS (2020). Improving the classification accuracy using recursive feature elimination with cross-validation. Int J Emerg Technol.

[52] Syarif I, Prugel-Bennett A, Wills G (2016). SVM parameter optimization using grid search and genetic algorithm to improve classification performance. Telkomnika.

[53] Zhang C, Ma Y (2012). Ensemble machine learning: methods and applications.

[54] Robin X, Turck N, Hainard A (2011). pROC: an open-source package for R and S+ to analyze and compare ROC curves. BMC Bioinformatics.

[55] Newcombe RG (1998). Two-sided confidence intervals for the single proportion: comparison of seven methods. Stat Med.

