# Supplementary material for: Hepatocellular carcinoma detection via targeted enzymatic methyl sequencing of plasma cell-free DNA
Source: Clin Epigenetics. 2023 Jan 4;15:2. doi: 10.1186/s13148-022-01420-6 (PMC9814445; doi:10.1186/s13148-022-01420-6)
Supplement: Supplementary file 1 — Additional file 1: Figure S1. Comparison of conversion efficiency between EM-seq and BS-seq. a Dot plot compare individual methylation values in hypermethylated CpGs acquired by EM-Seq and BS-seq (r = 0.91, P < 0.0001). Percentages indicate the fraction of CpGs that differed between conditions. Hypermethylated CpGs are those with BS-seq detection values of > 80% on pUC19 DNA. b Genome plot for unmethylated control λ DNA(30,000–35,000)compares CH reads between EM-seq and BS-seq datasets. Boxes represent reads, and unmethylated (blue) and methylated (red) CHGs are indicated. c Pie charts compare the proportion of reads with 0, 1, 2 and ≥ 3 CH sites in EM-seq (SRR10532128) and BS-seq (SRR10532135) sequencing reads from public database. d Methylation values on hypermethylated CpGs acquired by EM-Seq and BS-seq, before and after ≥ 3CHs filtration. ns represents no significance. e Dot plot compare individual methylation values in hypomethylated CpGs acquired by EM-Seq and BS-seq after 3CH read filtering. Percentages indicate the fraction of CpGs that differed between conditions. Figure S2. Performance of targeted EM-seq. a Unique read depth (PCR duplication removed) observed in EM-seq and BS-seq with same input quantities (20 ng). b Heat maps compare individual CpG methylation values in the target panel acquired by targeted EM-Seq between technical replicates (r = 0.999). c Heat map shows coverage depth of CpGs between replicates of a 20 ng targeted EM-seq library (r = 0.982). d Fragment size distributions of plasma sample from a healthy donor. Figure S3. Comparison of HCC predictive model with AFP and PIVKA-II. a Proportions of positive calling by HCC screening model in HCC patients with different AFP levels in the test set. b Proportions of positive calling by HCC screening model in HCC patients with different PIVKA-II levels in the test set. [file 13148_2022_1420_MOESM1_ESM.pptx]

## Slide 1
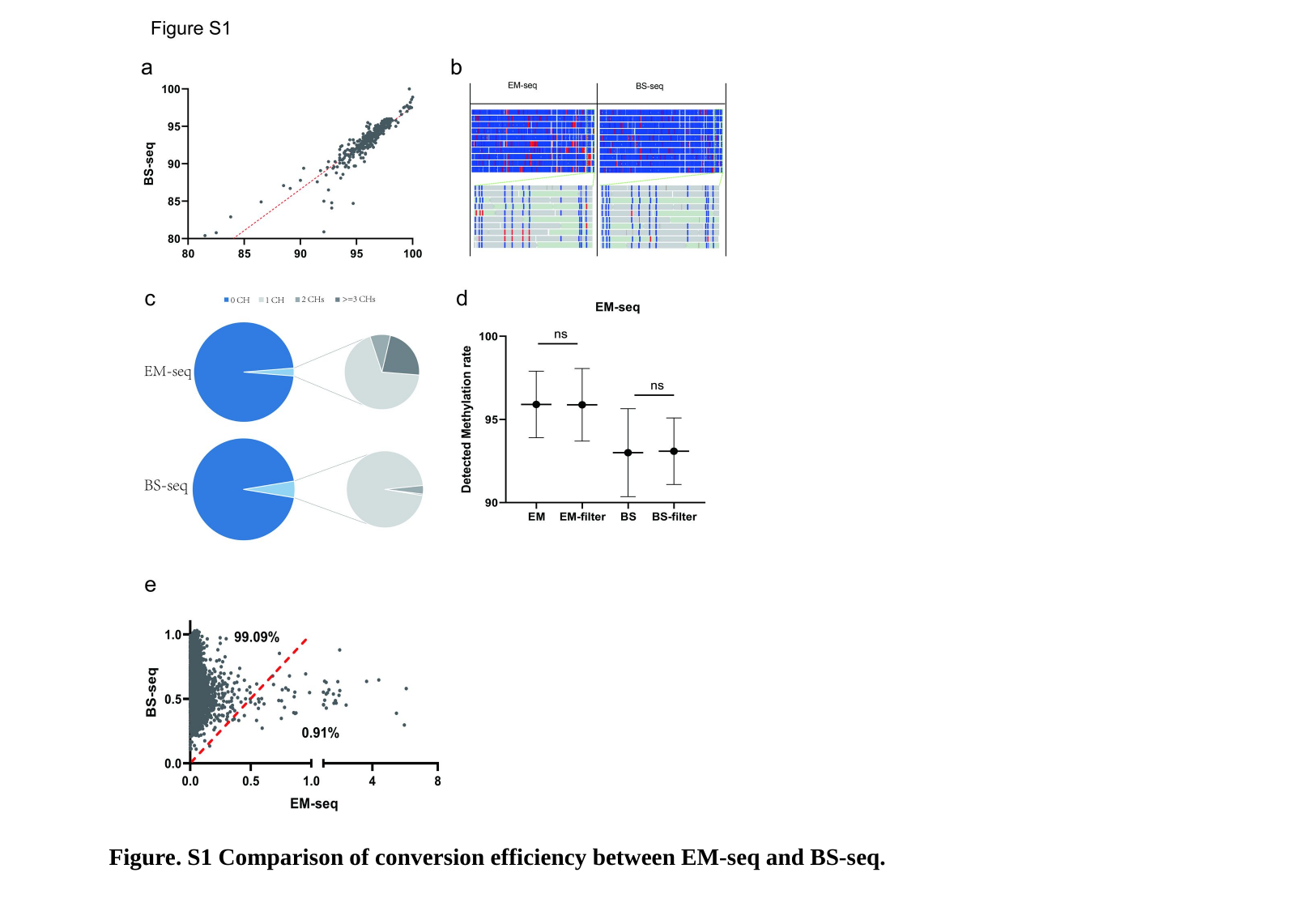

Figure. S1 Comparison of conversion efficiency between EM-seq and BS-seq.

## Slide 2
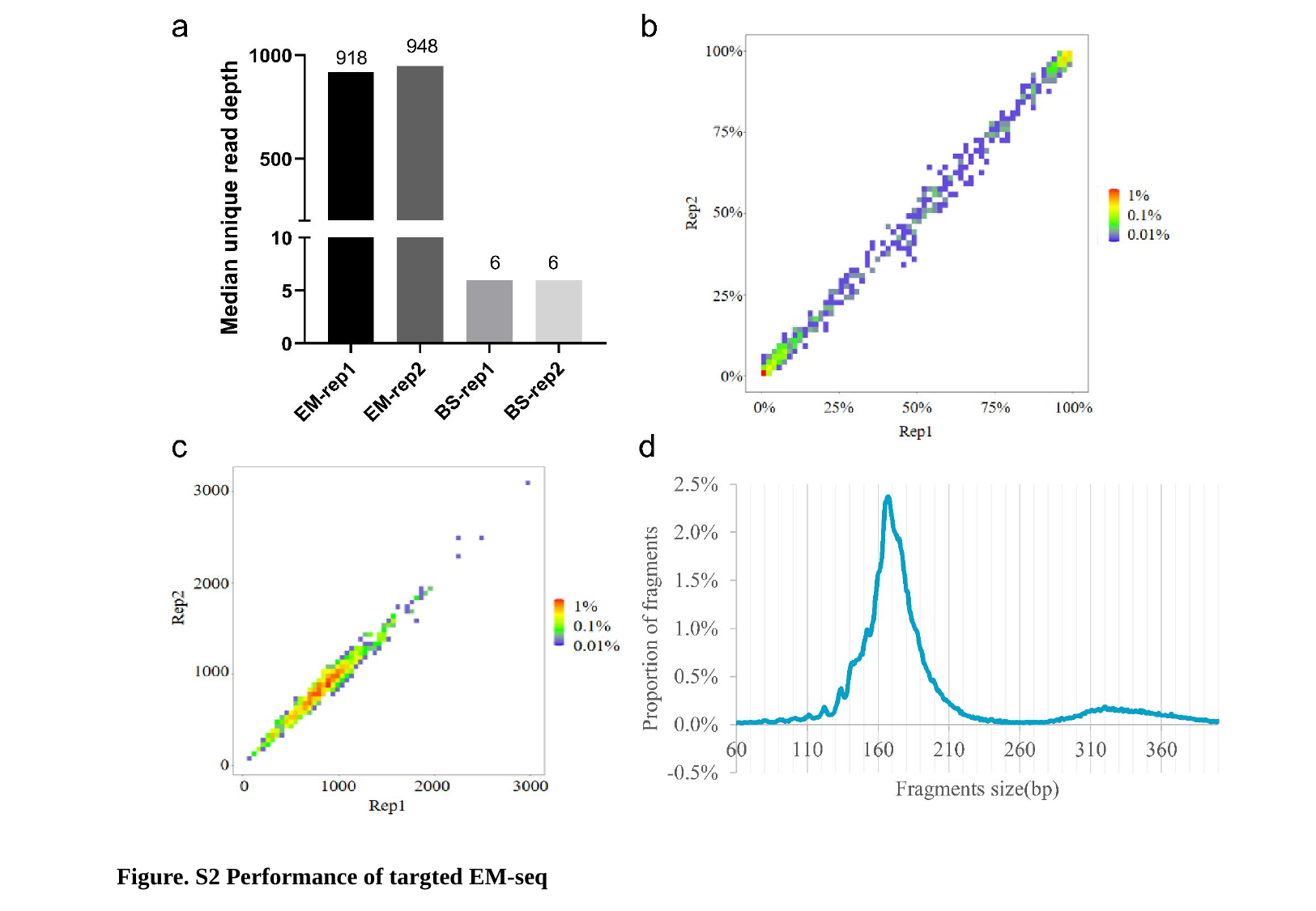

Figure. S2 Performance of targted EM-seq

## Slide 3
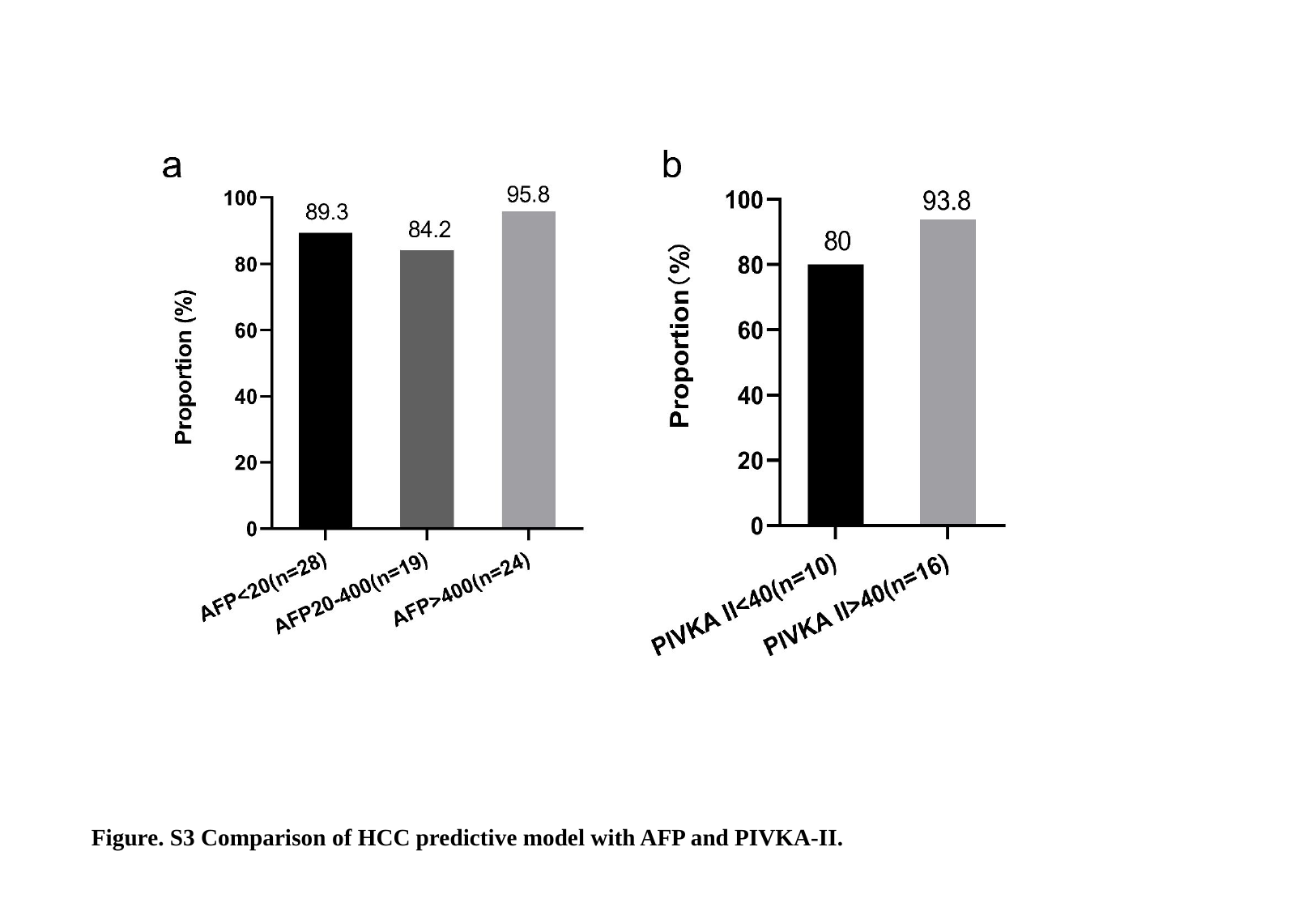

Figure. S3 Comparison of HCC predictive model with AFP and PIVKA-II.
